# Assessment of the Knowledge, Attitude, and Practice of Artificial Intelligence (AI) Among Radiologists, Physicians, and Surgeons in Saudi Arabia

**DOI:** 10.7759/cureus.79180

**Published:** 2025-02-17

**Authors:** Kholoud Sandougah, Nasser Alsywina, Hatan K Alghamdi, Faisal Alrobayan, Faisal M Alshaghathirah, Khaled Alomran

**Affiliations:** 1 Internal Medicine, College of Medicine, Imam Mohammad Ibn Saud Islamic University, Riyadh, SAU; 2 General Practice, Imam Mohammad Ibn Saud Islamic University, Riyadh, SAU; 3 College of Medicine, Imam Mohammad Ibn Saud Islamic University, Riyadh, SAU; 4 College of Medicine, Imam Mohammad Ibn Saud Islamic University, Saudi Arabia, Riyadh, SAU

**Keywords:** artificial intelligence, knowledge, pathology, physician, radiology

## Abstract

Introduction

Artificial intelligence (AI) is a broad term that refers to the idea and creation of computer systems capable of carrying out tasks that typically need human intelligence. Radiology, in particular, is becoming increasingly interested in the quick development of AI. There has been much discussion about the possible impacts of AI-based technologies on the development of radiology.

Aim

This study aimed to assess medical doctors' knowledge, attitudes, and practice regarding the applications of AI in radiology and other medical fields in Saudi Arabia.

Subject and methods

This cross-sectional study was conducted among medical doctors in Saudi Arabia. A self-administered questionnaire was distributed among the targeted doctors through the Saudi Commission of Health Specialties and an online survey using Google Forms. The survey comprised socio-demographic characteristics (i.e., age, gender, professional level, department unit) and questionnaires to assess the knowledge, attitude, and practice toward AI in the medical field.

Results

Of the 382 physicians, 56.8% were males and 71.7% were between 20 and 30 years old. Twenty-nine point six percent (29.6%) knew AI applications while only 12.3% were able to practice AI in the medical field. The overall attitude of physicians toward AI was positive among 26.2%. Increasing age and non-resident physicians were associated with better knowledge and attitudes toward AI application in the medical field. Lack of awareness (41.6%) and lack of proper training were recognized as the most common reasons for the reduced practice of AI in Saudi Arabia.

Conclusion

Despite optimistic attitudes, physicians' knowledge and practice toward AI application in the medical field were deficient. Younger residents were more likely to exhibit unfavorable knowledge and attitudes about AI while physicians working in the Internal Medicine unit were associated with poor practices. AI is innovating the medical field in various ways. Hence, further, larger studies are required to establish physicians' knowledge, attitudes, and practice regarding AI in the medical field.

## Introduction

One of the specialties of the field of computer science, known as artificial intelligence (AI), allows computers to do extremely complicated tasks with high accuracy by mimicking human cognitive processes like problem-solving [1]. AI is presently one of the areas of information technologies and computing that is growing the fastest, and it has the potential to have a significant impact on health care. The neural networks that form the basis of AI systems require large sets of data (also known as big data) for training [2]. High-quality labeled and representative real-life data are required to create machine learning or deep learning algorithms to prevent systematic bias. Failure to follow this fundamental rule could produce unreliable findings [3]. This is similar to how recommendations for the care of cancer patients were made based on the use of synthetic data [4]. Due to the availability of enormous volumes of data from the many diagnostic imaging modalities (such as X-ray, ultrasound, CT, MRI, and so on) that can be retrieved and used for training, radiology plays a crucial role in the development of AI algorithms. Therefore, it is anticipated that AI will have an impact not just on conventional radiological processes (such as image interpretation) but also on clinical decision support systems and structured reporting [2]. The practice of radiologists can be improved since AI-based tools can be used to accomplish laborious and repetitive activities and reading time more effectively [1]. To give radiologists a foundational understanding of AI, the European Society of Radiology (ESR) produced a white paper [5]. Additional uses for these advancements include the automatic identification of cases of pneumothorax, hemorrhage, kidney stones, and foreign substances in an emergency situation, aiding radiologists in the diagnosis process, speeding it up, and improving its accuracy [6]. According to several studies published in the literature, AI-based apps will not take the place of radiologists in their current roles; rather, they will enhance radiology services and radiologists' performance [1]. Human-machine interaction will become a crucial ability for all doctors and must be incorporated into medical education. The problem for medicine in the future is to enable quality checks when AI tools are utilized [3]. However, as the renowned Geoffrey Hinton, an authority on artificial neural networks, argues, the use of AI is a developing field in radiology, which can be concerning or even a threat to professional diagnostic radiologists [7] since teleradiology, 3D printing, implementing artificial intelligence in radiology, and other disciplines taking over radiological tests are heavily debated topics [6]. It is crucial to make sure that current clinical practitioners are knowledgeable about the status and potential of this technology in light of the ongoing AI revolution. Misinformation about how AI will impact clinical practice could result in unfavorable attitudes and ill-informed career decisions. Therefore, throughout this period of transition, it is crucial to give clinicians accurate, unbiased, and current information. In this perspective, it is critical to assess clinical doctors' feelings about AI's prospective applications. Therefore, the purpose of this study is to evaluate how physicians feel about using AI in radiology [8].

## Materials and methods

Study design

This is an observational, cross-sectional survey design study that attempts to assess the knowledge, attitude, and practice of AI in radiology and other medical fields among physicians in Saudi Arabia.

Study Setting

Electronic questionnaire targeting physicians who live in Saudi Arabia.

Sample Size

Saudi Arabian physicians are approximately 19,700 in number. There were 377 completed questionnaires ({Confidence Level: 95%} {Margin of error: 5%}).

Data Collection Methods

An electronic questionnaire targeting physicians who live in Saudi Arabia was used. The questionnaire includes four main sections, each concerning a different aspect of the objectives. All questions were reviewed by a radiologist consultant. The questionnaire has four main aspects:

Demographic information: The questionnaire includes questions on age, gender, qualification level, and rank.

Knowledge of artificial intelligence: This sub-scale has questions about the general knowledge of AI, including knowledge of artificial intelligence machine learning, AI in the medical field, AI in radiology, AI in pathology, and AI during the training for post-graduate doctors.

Attitude toward artificial intelligence: This sub-scale has questions about the attitude toward AI, including the necessity of AI in the medical field, training, assessment, diagnosis, radiology, and pathology.

Practice toward artificial intelligence: This sub-scale has questions about the practice of AI, including if the doctor has used AI in the medical field and the intention of using this technique during the training.

Data Analysis Plan

Data was stored in a Microsoft Excel data sheet (Microsoft Corporation, Redmond, WA, US), and upon completion of the data collection, it was exported to SPSS version 26 for further analysis.

Ethical approval

This study was approved by the Institutional Review Board at Al-Imam Muhammad Ibn Saud Islamic University, Riyadh, Saudi Arabia (Project number: 632/2024). All physicians gave their consent before taking part in this study.

Questionnaire criteria

Knowledge of AI in radiology and other medical fields was assessed using the question, "Do you know about any application of AI in the medical field?" while the question, "Have you ever applied AI technology in any field?" was used to assess the practice of AI in the radiology field. Both questions had "yes/no" answer options.

The attitude toward AI in radiology has been assessed using a 9-item questionnaire with a 5-point Likert scale category ranging from "no opinion" coded with 0 to "strongly agree" coded with 4. Negative questions were re-coded inversely to avoid bias in the score. The total attitude score was calculated by adding all 9 items. Scores ranging from 9 to 36 points were generated. The higher the score, the higher the attitude toward AI in the medical field. By using 50% and 75% as cutoff points to determine the level of attitude, physicians were classified as having a negative attitude if the score was below 50%, 50% to 75% were neutral, and above 75% were classified as having positive attitude levels.

## Results

Categorical variables were shown as numbers and percentages (%) while continuous variables were presented as mean and standard deviation. The relationship between knowledge and practice among the socio-demographic characteristics of physicians was conducted using the chi-square test. Further, the differences in the attitude score in relation to the socio-demographic characteristics of physicians were observed using the Mann-Whitney Z-test and Kruskal Wallis H-test. The normality test was conducted using the Kolmogorov-Smirnov test. The attitude score followed a non-normal distribution. Thus, the non-parametric tests were applied. Statistical significance was set to p<0.05 level. All data analyses were performed using SPSS version 26.

This study enrolled 382 physicians. Table 1 presents the socio-demographic characteristics of the physicians. The most common age group was between 20 and 30 years old, with more than half (56.8%) being males. Nearly three-quarters (73%) were residents, and 34.3% were working in the general surgical unit.

**Table 1:** Socio-demographic characteristics of the physicians (n=382)

Study Data	N (%)
Age group	
20 – 30 years	274 (71.7%)
31 – 40 years	65 (17.0%)
41 – 50 years	33 (08.6%)
51 – 60 years	09 (02.4%)
>60 years	01 (0.30%)
Gender	
Male	217 (56.8%)
Female	165 (43.2%)
Professional level	
Resident	279 (73.0%)
Registrar	44 (11.5%)
Consultant	45 (11.8%)
Assistant professor	04 (01.0%)
Associated professor	03 (0.80%)
Professor	05 (01.3%)
General Practioner	02 (0.50%)
Departmental unit	
Internal medicine unit	116 (30.4%)
General surgery	131 (34.3%)
Family or emergency medicine unit	40 (10.5%)
Pediatric or dentistry	57 (14.9%)
Other allied unit	38 (09.9%)

Regarding the assessment of the knowledge of AI in radiology and other medical fields, nearly all (93.7%) knew the meaning of AI while their knowledge of AI subtypes was deficient (18.8%). Only 29.6% were aware of AI applications in the medical field, and only 12.3% had learned about AI in medical school. Also, physician's knowledge of AI applications in radiology and pathology was deemed poor (18.8% and 11%, respectively). In addition, only 11.5% indicated that the AI curriculum was included in their training. When assessing the attitude of AI in radiology and other medical fields, it was observed that the top three statements with the highest ratings were, "Believe that the physician's role is important in applying and evaluating AI in the medical field" (mean score: 2.96), followed by "Believe that AI should be included in the curriculum in medical school as well as specialist training" (mean score: 2.95), and "Believe AI is essential in the medical field" (mean score: 2.85). Based on 9 attitude items, the overall mean attitude score was 23.4 (SD 7.06), with negative, neutral, and positive attitude levels constituting 12.8%, 61%, and 26.2%, respectively (Table 2).

**Table 2:** Assessment of the knowledge and attitude of AI in radiology and other medical fields (n=382) † Reverse-coded question Attitude items have a response ranging from "no opinion" coded with 0 to "strongly agree" coded with 4.

Knowledge items	N (%)
Do you know what is artificial intelligence?	
Yes	358 (93.7%)
No	24 (06.3%)
Do you know about machine learning and deep learning (subtypes of AI)?	
Yes	72 (18.8%)
No	310 (81.2%)
Do you know about any application of AI in the medical field?	
Yes	113 (29.6%)
No	269 (70.4%)
Have you ever been taught about artificial intelligence in medical school?	
Yes	47 (12.3%)
No	335 (87.7%)
Do you know about the application of AI in radiology?	
Yes	72 (18.8%)
No	310 (81.2%)
Do you know about the application of AI in the pathology field?	
Yes	42 (11.0%)
No	340 (89.0%)
Did your training include a curriculum regarding AI?	
Yes	44 (11.5%)
No	338 (88.5%)
Attitude items	Mean ± SD
Believe that the physician's role is important in applying and evaluating AI in the medical field	2.96 ± 1.17
Believe that AI should be included in the curriculum in medical school as well as specialist training	2.95 ± 0.97
Believe AI is essential in the medical field	2.89 ± 0.96
Believe that AI aids practitioners in early diagnosis and assessment of the severity of disease	2.85 ± 0.97
Believe that AI will replace physicians in the future ^†^	2.58 ± 1.14
Believe AI is very essential in the field of radiology	2.36 ± 1.28
Believe AI is essential in the field of Pathology	2.27 ± 1.33
Believe AI would be a burden for practitioners ^†^	2.31 ± 1.18
Believe AI would increase the percentage of errors in diagnosis	2.22 ± 1.01
Total attitude score	23.4 ± 7.06
Level of attitude	
Negative	49 (12.8%)
Neutral	233 (61.0%)
Positive	100 (26.2%)

In terms of practice, more than half (51%) intend to work on AI in the future; however, only 12.3% have ever used AI technology in any field (Table 3). Among those who previously used AI (N=47), the most common imaging modality where AI was being applied was X-ray (29.8%); for pathology assessment, microcopy was more common (10.6%). Also, 46.8% and 48.9% indicated that AI applications are easy to use and make tasks easy.

**Table 3:** ‎Assessment of practice of AI in radiology and other medical fields (n=382)

Practice items	N (%)
Would you like to work on AI in the future?	
Yes	195 (51.0%)
No	61 (16.0%)
I don't know	126 (33.0%)
Have you ever applied AI technology in any field?	
Yes	47 (12.3%)
No	335 (87.7%)
Which radiographic modalities have you used for AI applications? (n=47)	
No Any	15 (31.9%)
X-ray	14 (29.8%)
CT scan	10 (21.3%)
MRI	05 (10.6%)
PET scan	02 (04.3%)
Others	01 (02.1%)
For which pathological assessment you have used AI? (n=47)	
None	32 (68.1%)
Histopathology	03 (06.4%)
Culture sensitivity	03 (06.4%)
Microscopy	05 (10.6%)
Frozen section	03 (06.4%)
Ultrasound	01 (02.1%)
Was it easy for you to apply AI? (n=47)	
Yes	22 (46.8%)
No	25 (53.2%)
Did AI make your task easy? (n=47)	
Yes	23 (48.9%)
No	24 (51.1%)

**Figure 1:**
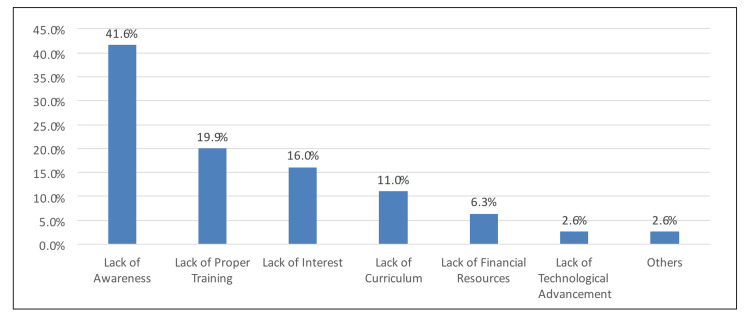
Perceived reason for the reduced practice of AI in Saudi Arabia

Figure 1 depicts that the most common reason for the reduced practice of AI was lack of awareness (41.6%), followed by lack of proper training (19.9%) and lack of interest (16%).

Measuring the relationship between the knowledge and practice of AI in radiology according to the socio-demographic characteristics of the physicians found that the increasing age (p<0.001) and non-residents (p<0.001) were more associated with having the knowledge of AI in the medical field while working in other allied units (i.e., family medicine, emergency medicine, etc.) were associated with having both the knowledge and practice of AI in the medical field (p<0.05). No significant relationships were observed between the knowledge and practice of AI in medical fields in terms of gender (p>0.05) (Table 4).

**Table 4:** Relationship between the knowledge and practice of AI in radiology among the socio-demographic ‎characteristics of the physicians (n=382)‎ § P-value has been calculated using the chi-square test. ** Significant at the p<0.05 level.

Factor	Knowledge of any application of AI in the medical field		X2	P-value ^§^	Application of AI		X2	P-value ^§^
	Yes, N (%) (n=113)	No, N (%) (n=269)			Yes, N (%) (n=47)	No, N (%) (n=335)		
Age group								
≤30 years	62 (54.9%)	212 (78.8%)	22.495	<0.001**	29 (61.7%)	245 (73.1%)	2.656	0.103
>30 years	51 (45.1%)	57 (21.2%)			18 (38.3%)	90 (26.9%)		
Gender								
Male	60 (53.1%)	157 (58.4%)	0.900	0.343	23 (48.9%)	194 (57.9%)	1.353	0.245
Female	53 (46.9%)	112 (41.6%)			24 (51.1%)	141 (42.1%)		
Professional level								
Resident	63 (55.8%)	216 (80.3%)	24.344	<0.001**	31 (66.0%)	248 (74.0%)	1.364	0.243
Non-resident	50 (44.2%)	53 (19.7%)			16 (34.0%)	87 (26.0%)		
Departmental unit								
Internal medicine unit	22 (19.5%)	94 (34.9%)	15.969	<0.001**	09 (19.1%)	107 (31.9%)	7.741	0.021**
General surgery	35 (31.0%)	96 (35.7%)			13 (27.7%)	118 (35.2%)		
Other allied unit	56 (49.6%)	79 (29.4%)			25 (53.2%)	110 (32.8%)		

Exploring the association between the attitude toward AI in the medical field and the socio-demographic characteristics of the physicians found that increasing attitude scores were associated with increasing age (Z=3.627; p<0.001) and non-residents (Z=2.677; p=0.007). No significant differences were observed between the attitude toward AI in the medical field in terms of gender (p=0.148) and departmental unit (p=0.925) (Table 5).

**Table 5:** Association between the attitude of AI in radiology and the socio-demographic characteristics of the ‎physicians (n=382) § P-value was calculated using the Mann-Whitney Z-test. ‡ P-value was calculated using the Kruskal Wallis H-test. ** Significant at the p<0.05 level.

Factor	Attitude Score (36) Mean ± SD	Z-test	P-value ^§^
Age group			
≤30 years	22.7 ± 7.28	3.627	<0.001 **
>30 years	25.0 ± 6.18		
Gender			
Male	23.8 ± 6.79	1.448	0.148
Female	22.9 ± 7.37		
Professional level			
Resident	22.8 ± 7.39	2.677	0.007 **
Non-resident	24.9 ± 5.84		
Departmental unit			
Internal medicine unit	23.6 ± 6.22	0.157	0.925 ^‡^
General surgery	23.2 ± 7.35		
Other allied unit	23.4 ± 7.47		

## Discussion

This study explored physicians' knowledge, attitudes, and practices (KAP) regarding AI in the medical field. The findings of this study will be a great addition to the literature, given the increasing trend of AI use in the medical field, particularly in the radiology field, where it is known to enhance the accuracy and efficiency of interpreting medical images (i.e., X-rays, MRIs, CT scans) [9]. Thus, this study would provide more data and determine whether physicians in Saudi Arabia are equipped with adequate KAP on AI.

Knowledge

Our study's results identified gaps in AI knowledge. Despite most physicians being aware of AI in general, their knowledge of its application in the medical field was low (29.6%). Also, their understanding of the application of AI in radiology and pathology showed poor ratings. In addition, neither teaching AI in medical school nor attending training achieved adequate results. This indicates that physicians in our region need more education and training to improve their understanding of AI in the medical field. Consistent with our reports, several studies suggest that physicians, medical students, and radiologist professionals demonstrated basic knowledge of AI, but their knowledge of its application in the medical field was suboptimal [10-14]. A systematic review by Mousavi Baigi et al. (2023) also supported these reports, with only half of students being aware of AI [15]. There is clear evidence that AI was not fully explored in the medical field. Awareness, training, and practical exposure are necessary to improve the population's understanding and increase interest in integrating AI into the medical field.

Significant Factor of Knowledge

The knowledge of AI varied significantly according to age, professional level, and department unit. In particular, older physicians, non-residents (i.e., consultants, professors, registrars, etc.), and working with other department units were associated with better knowledge of AI. Non-resident physicians, such as consultants, professors, and registrars, had more experience than the resident physicians while the differences in AI knowledge between department units could be due to different training and specialty areas of interest. However, both specialties can benefit from AI in the medical field. On the contrary, a study by Huang et al. (2024) found that higher education, increasing years of radiology experience, AI-diagnosis-related workshops, and involvement in research related to AI diagnosis were more likely to have sufficient knowledge of AI [16]. However, literature published by Al-Medfa et al. (2023) reported no association between the participants' gender and years of experience in relation to AI knowledge [17]. Our study also found no significant relationship between AI knowledge and gender, which is consistent with previous reports.

Attitude

The overall attitude of physicians regarding AI was favorable. According to our results, more than one-quarter of our subjects were deemed to have a positive attitude toward AI. Only 12.8% were considered negative attitudes (mean score: 23.4 out of 36 points). This is consistent with the study done among residents [2], students [15], and physicians [17]. However, a study conducted in Riyadh showed that even though radiologists showed a positive attitude toward AI, some radiologists had the wrong perception that AI might replace their jobs [14]. AI has the ability to improve and transform various aspects of the medical field, but it is doubtful that it will completely replace humans in the future [18]. However, AI capabilities could be incorporated with healthcare professionals' skills to improve accuracy, efficiency, and quality of care in the medical field [19].

Significant Factor of Attitude

Data from this study suggest that older non-resident physicians were more likely to exhibit better attitudes toward AI. This does not agree with the study of Huang et al. (2024). Positive attitudes varied significantly according to education, years of experience in radiology, and AI diagnosis-related training [16]. This corroborates the report of Swed et al. (2022). Sixth-year students were more likely to have a positive attitude toward AI than first-year students [12]. However, a paper by Qurashi et al. (2021) found no significant differences between the level of AI agreement in relation to profession, years of experience, academic qualification, and hospital type [1]. In our study, we also found no significant differences between gender and department units in relation to attitudes (p>0.05). The differences in results could be due to the type of population, study methodology, and population size. Hence, these findings necessitated further investigations to determine the factors influencing attitudes toward AI in the medical field.

Practice

More than half of our subjects have shown interest in working with AI in the future; however, the actual application of AI technology in their practice was seen to be low (12.3%). Among those who previously used AI radiology, about one-third of them used AI to read X-rays and CT scans. However, in pathology assessment, the use of AI-aided technology was uncommon. Overall, their confidence in using AI was less than desired. This contradicted the reports conducted among Sudanese physicians. Approximately 80% showed interest in using AI in the future, and nearly two-thirds of the physicians had good practice with AI [11]. However, a study published in Bahrain reported that pathologists backed the use of AI in their field, stating that AI could "Formulate personalized medication and/or treatment plans for patients" and "Interview patients in a range of settings to obtain a medical history." Most participants believed that AI would decrease the time needed for diagnostic tests and may not affect employment rates [17].

Moreover, lack of awareness, proper training, interest, curriculum, and financial resources are some of the most common barriers to using AI in the medical field. This is strikingly similar to the study conducted in Pakistan. The primary reasons for failing to implement AI were a lack of knowledge and awareness, lack of interest in AI, lack of training and curriculum, inadequate financial resources, and poor technological advancements [14]. However, among Italian resident physicians, potential AI issues were identified such as the risk of a lower professional reputation of radiologists and higher costs and workload due to the system maintenance of AI [2]. Addressing these barriers is important to increase AI utilization in the medical field and improve patient care and clinical outcomes.

Significant Factor of Practice

Findings suggest that physicians working in other department units were associated with increased use of AI. This is not consistent with the literature published in Syria. The study indicated that the practice of AI varies significantly by age and qualification [12]. Our study observed no significant relationships between the use of AI in terms of age, gender, and professional level (p>0.05), which did not coincide with previous reports. Further investigations are required to confirm these findings.

Study limitations

This study found some limitations. First, most of the physicians were young and residents; thus, we cannot generalize the pairwise comparison of age and professional level in relation to AI KAP. Second, the convenience sampling method could result in sampling bias, leading to skewed data or non-representative findings. Third, online surveys may be prone to answer bias, resulting in some data quality issues. Lastly, a cross-sectional survey could be prone to bias, unable to determine cause and effect, and cannot be used to examine behavior over time.

## Conclusions

Physicians' attitudes toward AI in the medical field were better than their knowledge and practices. Older nonresident physicians were associated with a better attitude, but physicians working with internal medicine units were linked to poor knowledge and practices toward AI. Lack of awareness, lack of proper training, and lack of interest were identified as detrimental factors for increased practice of AI. The knowledge and practices regarding AI can be improved by addressing the barriers. It requires a multifaceted approach, which can be done through collective efforts by healthcare authorities, providers, AI developers, and policymakers, enabling the conscientious implementation of AI in the medical field.
